# Delegates to the Convention for Revising the United States Pharmacopœia

**Published:** 1850-02

**Authors:** 


At a meeting of the College of Physicians of Philadelphia, held January 15th, 1850, the following Fellows were elected Delegates to the Convention for Revising the United States Pharmacopœia:

Joseph Carson, Henry Bond, Francis West.

